# Performance of two questionnaires to measure treatment adherence in patients with Type-2 Diabetes

**DOI:** 10.1186/1471-2458-9-38

**Published:** 2009-01-26

**Authors:** Carlos A Prado-Aguilar, Yolanda V Martínez, Yolanda Segovia-Bernal, Rosendo Reyes-Martínez, Raul Arias-Ulloa

**Affiliations:** 1 Unidad de Investigación Epidemiológica y en Servicios de Salud, Hospital General de Zona N° 1, Instituto Mexicano del Seguro Social, Aguascalientes, Ags., México; 2 Unidad de Medicina Ambulatoria, Instituto Mexicano del Seguro Social, Aguascalientes, Ags., México; 3 Unidad de Medicina Familiar, Instituto Mexicano del Seguro Social, Aguascalientes, Ags, México; 4 Depto. de Salud Publica, Universidad Autónoma de Aguascalientes, Aguascalientes, Ags, México

## Abstract

**Background:**

Most valid methods to measure treatment adherence require time and resources, and they are not easily applied in highly demanding Primary Health Care Clinics (PHCC). The objective of this study was to determine sensitivity, specificity, predictive values, likelihood ratios, and post-test probabilities of two novel questionnaires as proxy measurements of treatment adherence in Type-2 diabetic patients.

**Methods:**

Two questionnaires were developed by a group of experts to identify the patient's medical prescription knowledge (knowledge) and their attitudes toward treatment adherence (attitudes) as proxy measurements of adherence. The questionnaires were completed by patients receiving care in PHCC pertaining to the Mexican Institute of Social Security in Aguascalientes (Mexico). Pill count was used as gold standard. Participants were selected randomly, and their oral hypoglycemic prescriptions were studied. The main outcome measures for each questionnaire were sensitivity, specificity, predictive values, likelihood ratios, and post-test probabilities, all as an independent questionnaire test and in a serial analysis.

**Results:**

Adherence prevalence was 27.0% using pill count. Knowledge questionnaire showed the highest sensitivity (68.1%) and negative predictive value (82.2%), the lowest negative likelihood ratio (0.58) and post-test probability for a negative result (0.16). Serial analysis showed the highest specificity (77.4%) and positive predictive value (40.1%) as well as the highest positive likelihood ratio (1.8) and post-test probability for a positive result (0.39).

**Conclusion:**

Medical Prescription Knowledge questionnaire showed the best performance as proxy measurement to identify non-adherence in type 2 diabetic patients regarding negative predictive value, negative likelihood ratio, and post-test probability for a negative result. However, Medical Prescription Knowledge questionnaire performance may change in contexts with higher adherence prevalence. Therefore, more research is needed before using this method in other contexts.

## Background

In the clinical guidelines for the care of patients with Type-2 Diabetes Mellitus (DM2), the Mexican Institute of Social Security (Instituto Mexicano del Seguro Social, IMSS) establishes the evaluation of treatment adherence as one of the 29 care actions [1]. This is motivated by the fact that treatment adherence in diabetic patients is associated with lower HbA1c levels [2] and a decrease in the risk of complications [3].

Direct methods used to measure treatment adherence include the measurement of drug serum concentration, or the use of chemical markers in tablets. However, these methods are expensive and they also have known limitations [4], restricting their daily use in primary health care clinics (PHCC). Electronic monitoring is one of the most commonly used indirect methods, and it is the closest method to a gold standard [5]. Such electronic monitoring provides detailed information about the use of medications, keeping records of dates and hours at which the bottles were opened. While its usefulness has been demonstrated in patients who require long-term treatment [5-7], the expense of this method in clinical settings is a serious drawback. Pill counts require at least two home visits to the patient [6,7], which also makes this impractical in a high-demand PHCC setting. Other indirect methods such as clinical outcomes [8] and patient self-reports tend to overestimate adherence, unlike electronic monitoring [6].

A questionnaire to measure adherence has been developed, based on the level of disease knowledge [9]. However, this questionnaire tends to underestimate adherence prevalence and while its sensitivity is adequate, it has a low specificity and negative predictive value [10,11]. Similarly, a questionnaire to measure adherence was developed on the basis of behavioral attitudes regarding the intake of medication [12], but this tends to overestimate adherence prevalence and between 47 and 67% of patients are misclassified as adherent [10,11].

The objective of this study was to determine the sensitivity, specificity, predictive values, likelihood ratios, and post-test probabilities of two novel questionnaires as a proxy measurement of treatment adherence in Type-2 diabetic patients.

## Methods

### Development of the questionnaires

A group of experts developed two questionnaires as proxy measurements of adherence through the identification of patient's medical prescription knowledge (knowledge) and attitudes toward treatment adherence (attitudes) using a consensus-panel process. The questionnaires were written in Spanish. The first phase of development included item generation by diabetologists, primary-care physicians, and psychologists. In the first questionnaire, three questions were formulated that assessed the patient's knowledge of the name of the medication prescribed to control their diabetes (KQ1), its dosage (KQ2), and the dosing interval (KQ3). The second questionnaire addressed six domains that were identified by the experts in a thorough search for information about the causes of nonadherence: well-being/discomfort (AQ1, AQ2); belief about the damage caused by medication (AQ3); the diabetes-treatment complications relationship (AQ4, AQ5); barriers/facilitators to take medication (AQ6, AQ7); accessibility to healthcare and medical treatment (AQ8, AQ9); and doctor-patient agreements about treatment (AQ10, AQ11). Experts developed eleven questions, two of which were reworded from the Morisky scale [12]. The experts agreed that both questionnaires had good content validity.

The second phase of questionnaires development was face validity. Patients understanding of questions on written and oral formats were explored. As reading problems were identified, interviews were used for data collection. These questionnaires were completed by a convenience sample of 30 diabetic patients. They were asked to identify ambiguous items. Based on their feedback, some of the questions were reworded to eliminate ambiguous phrasing.

The final phase was done in order to provide support for the construct validity of the attitudes questionnaire. We carried out a principal component analysis followed by a varimax rotation, to explore if the attitudes questionnaire domains were valid. An item was considered to be correlated on a domain if factor loading was greater than 0.4 [13]. This analysis yielded six factors with eigenvalues > 1, explaining 77.5% of the variance [see Additional file 1]. Therefore, the questionnaire structure identified by the experts through literature was confirmed, and it showed construct validity. Further, a Cronbach's alpha was used to examine the internal consistency of the attitude questionnaire, which was acceptable (alpha = 0.74).

Each question of attitudes scale was rated on a five-point (1–5) Likert scale of agreement, ranging from "strongly disagree" to "strongly agree". Questionnaires' English version [see Additional file 2] is a translation from the authors, and had not been adapted to this language; the originals Spanish questionnaires are available from the first author.

### Context and participants

In Mexico, IMSS provides health services to more than 50% of the population; diabetes was the second reason for consultation in primary health care in 2001. Type-2 diabetes accounts for over 90% of the cases. This study was carried out in PHCC situated in the urban areas of Aguascalientes, Mexico.

Participants were randomly selected from a Register of Chronic Degenerative Patients in the IMSS; this database had approximately 27,850 patients with diabetes in Aguascalientes during 2001.

Inclusion criteria for all participants were: patients ≥40 years of age, who had been diagnosed with DM2 at least one year earlier, who received oral hypoglycemic medication (glibenclamide and/or metformin), and who did not use insulin or suffer from chronic complications.

We calculated the sample size taking into account each oral hypoglycemic prescribed. Due to some diabetic patients having monotherapy or polytherapy prescription, the number of medications in our study is larger than the number of patients. Further, we took into account the prevalence of adherence reported by Donnan et al. [14] in patients with polytherapy (35%). With this prevalence being low, it is expected that the questionnaires are able to detect most of the patients who do not adhere [15]. As a result, the sample size calculation was done to identify a specificity of 80% to detect nonadherence (95% confidence level) and a precision of 5%. The sample size was of 379 medications to study. EPIDAT 3.1 [16] was used to calculate the size and power of the sample, which uses the proposed formulae by Obuchowsky N [17] for studies of test accuracy.

### Data collection

Two health professionals were trained to carry out home visits and patient interviews. In the PHCC, the interviewers invited the patients to participate and explained the details of their participation in the research by means of an informed consent form.

In the first home visit, interviewers recorded the number of pills that patients had received, and also completed the questionnaires about personal characteristics, medical prescription knowledge, and attitudes to treatment adherence. Over three months and each month, interviewers visited the patients again to register the pills that patients received in their consultation; at the last visit, they registered the pill count.

The family physician's prescriptions were verified from medical records at the PHCC (type of oral hypoglycemic, dosage, and dosing interval).

### Statistical analysis

The true level of treatment adherence was identified by means of the pill count. As a result, this method gave the percentage of adherence which was calculated by dividing the difference in the number of pills in the first home visit, and the pills remaining at the last home visit by the number of pills prescribed for the time interval, and multiplying the result by 100. Pill count percentages were converted to a categorical scale, as proposed by Mason [6]. As such, a patient who took between 90 and 105% of the medication prescribed was classified as having good adherence, and a patient that took < 90% or > 105% was classified as having poor adherence.

### Proxy measurements of treatment adherence

To classify attitudes, answers to the questionnaire were added and divided by the number of items. A result of 4 or 5 was classified as a positive attitude and a result of 1–3 was classified as a negative attitude. A patient was classified as having strong knowledge when the answer to the three questions matched the physician's prescription. When at least one answer did not match with physician's prescription the patient was classified as having weak knowledge about the medical prescription.

The questionnaires of attitudes and knowledge were combined for a serial analysis [18]. This analysis considered a patient with a positive attitude and strong medical prescription knowledge as having good adherence, and a patient with a negative attitude or weak medical prescription knowledge was classified as having poor adherence.

Descriptive statistics were calculated for the sociodemographic and clinical characteristics of Type-2 diabetic patients and for treatment adherence, knowledge, attitudes, and serial analysis. Continuous data with normal distribution were described using mean and its standard deviation, or median and quartiles for variables without normal distribution. These data analyses were conducted using SPSS 11.0 [19].

Principal component analysis and the internal consistency of the attitudes questionnaire were evaluated using SPSS [19]. The Medcalc statistical software, v10.0.1 [20] was used to calculate the sensitivity, specificity, predictive values, likelihood ratios, and post-test probabilities for each questionnaire in independent analysis, and for a serial analysis. This study was approved by the IMSS Research Committee in Aguascalientes.

## Results

Patients responded to interviews about sociodemographic characteristics, knowledge, and attitudes questionnaires in a mean time of 10 min.

Sociodemographic and clinical characteristics and adherence percentages are shown in Table 1. Over a half of patients were female 62.2%, most of them had basic education 79.4%, and were married or lived in free union 76.5%. Mean age was 58.7 ± 9.6. More than half the participants suffered hypertension 63.0% and had polytherapy prescription 66.0%. The median of duration of diabetes since first diagnosis was 6 years and median level of fasting glucose was 8.8 mmol/L, mean HbA1c was 8.8% ± 2.3.

**Table 1:** Characteristics of Type-2 diabetic patients.

CHARACTERISTICS		Type-2 diabetic patients n = 238
Sociodemographic characteristics Gender	n (%)	
Female		148 (62.2)
Male		90 (37.8)
Education level (years)	n (%)	
Basic education (6c)		189 (79.4)
Intermediate level (7 to 9)		24 (10.1)
Higher education (10≥)		25 (10.5)
Civil status	n (%)	
Singled		13 (5.5)
Married/Free union		182 (76.5)
Divorced/Widow (er)		43 (18.0)
Age	mean (SD)	58.7 (9.6)
Clinical characteristics		
Hypertension	n (%)	
Yes		150 (63.0)
No		88 (37.0)
Prescription	n (%)	
Monotherapy		81 (34.0)
Polytherapy		157 (66.0)
Duration of diabetes in years (since first diagnosis)	median (quartiles)	6 (3–12)
Fasting glucose mmol/L	median (quartiles)	8.8 (6.9–11.9)
HbA1c %	mean (SD)	8.8 (2.3)
		Medications n = 407
Treatment adherence	n (%)	
Good		110 (27.0)
Poor		297 (73.0)
Medical prescription Knowledge	n (%)	
Strong		210 (51.6)
Weak		197 (48.4)
Attitude to treatment Adherence	n (%)	
Positive		207 (50.9)
Negative		200 (49.1)
Combination of knowledge and attitude	n (%)	
Good		112 (27.5)
Poor		295 (72.5)

Approximately, a quarter of patients showed good adherence 27.0%. Proxy measurements of adherence as serial analysis classified patients with good adherence when they had a strong knowledge and positive attitude, with 27.5%. Considering knowledge and attitudes separately, their percentages were similar; strong knowledge was 51.6% and positive attitudes was 50.9%.

Table 2 shows the performance of the Medical Prescription Knowledge questionnaire; 68.1% of the patients with good treatment adherence were detected by the questionnaire (sensitivity = 68.1%, 95% C.I. 58.6 – 76.6), 82.2% of the patients with weak medical prescription knowledge had poor treatment adherence (negative predictive value 82.2%, 95% C.I. 76.1–87.3), patients with poor adherence are 0.58 times more likely to have weak medical prescription knowledge than patients with good adherence (negative likelihood ratio = 0.58, 95% C-I- 0.44–0.78). If a patient had weak medical prescription knowledge, the patient's probability of having good treatment adherence reduces from 27% to 16% (Post-test probability of a negative result = 0.16 95% C.I. 0.10 – 0.25).

**Table 2:** Medical prescription knowledge as a proxy measurement of treatment adherence with pill count as gold standard.

MEDICAL PRESCRIPTION KNOWLEDGE	TREATMENT ADHERENCE		
		
	Good	Poor	
Strong	75	135	210
Weak	35	162	197

	110	297	407

Sensitivity (95% CI): 68.1% (58.6–76.7)

Specificity (95% CI): 54.5% (48.6–60.3)

Positive predictive value (95% CI): 35.7% (29.2–42.6)

Negative predictive value (95% CI): 82.2% (76.1–87.3)

Positive likelihood ratio (95% CI): 1.50 (1.25–1.79)

Negative likelihood ratio (95% CI): 0.58 (0.44–0.78)

Pre-test odds (95% CI): 0.36 (0.28–0.44)

Positive post-test odds (95% CI): 0.54 (0.35–0.78)

Positive post-test probability (95% CI): 0.35 (0.25–0.43)

Negative post-test odds (95% CI): 0.20 (0.12–0.34)

Negative post-test probability (95% CI): 0.16 (0.10–0.25)

Results of the performance of the attitudes toward treatment adherence scale are shown in table 3. These results were no better than those showed by the Medical Prescription Knowledge questionnaire. Although, questionnaires serial analysis showed an adequate specificity (77.4%, 95% C.I. 72.2–82.0) and a good negative predictive value (77.9%, 95% C.I. 72.8–82.5), the overall performance of the serial analysis (table 4) did not exceed the performance of the Medical Prescription Knowledge questionnaire.

**Table 3:** Attitude toward treatment adherence as a proxy measurement of Treatment adherence with pill count as gold standard.

ATTITUDES TOWARD TREATMENT ADHERENCE	TREATMENT ADHERENCE		
		
	Good	Poor	
Positive	63	144	207
Negative	47	153	200

	110	297	407

Sensitivity (95% CI): 57.2% (47.4–66.6)

Specificity (95% CI): 51.5% (45.6–57.3)

Positive predictive value (95% CI): 30.4% (24.2–37.1)

Negative predictive value (95% CI): 76.5% (70.0–82.1)

Positive likelihood ratio (95% CI): 1.18 (0.97–1.44)

Negative likelihood ratio (95% CI): 0.83 (0.65–1.06)

Pre-test odds (95% CI): 0.36 (0.28–0.44)

Positive post-test odds (95% CI): 0.42 (0.27–0.63)

Positive post-test probability (95% CI): 0.29 (0.21–0.38)

Negative post-test odds (95% CI): 0.29 (0.18–0.46)

Negative post-test probability (95% CI): 0.22 (0.15–0.31)

**Table 4:** Serial analysis as a proxy measurement of treatment adherence with pill count as gold standard.

SERIAL ANALYSIS	TREATMENT ADHERENCE		
		
	Good	Poor	
Good	45	67	112
Poor	65	230	295

	110	297	407

Sensitivity (95% CI): 40.9% (31.6–50.6)

Specificity (95% CI): 77.4% (72.2–82.0)

Positive predictive value (95% CI): 40.1% (31.0–49.8)

Negative predictive value (95% CI): 77.9% (72.8–82.5)

Positive likelihood ratio (95% CI): 1.8 (1.33–2.47)

Negative likelihood ratio (95% CI): 0.76 (0.65–0.90)

Pre-test odds (95% CI): 0.36 (0.28–0.44)

Positive post-test odds (95% CI): 0.64 (0.37–1.08)

Positive post-test probability (95% CI): 0.39 (0.27–0.51)

Negative post-test odds (95% CI): 0.27 (0.18–0.39)

Negative post-test probability (95% CI): 0.21 (0.15–0.28)

## Discussion

### Main findings

This study evaluated whether the knowledge questionnaire and attitude scale can be used as proxy measurements of treatment adherence in Type-2 diabetic patients, using pill count as gold standard.

The study population showed low adherence prevalence (27.0%), therefore, it is important to explore possible consequences when adherence prevalence is low. In this situation, most of the population will not adhere to their prescriptions, and specific questionnaires might be expected to be capable of detecting most of the patients that do not adhere [15]. In this context it is important to detect patients who do not adhere in order to commence timely interventions to improve their adherence.

The best performance was identified with the Medical Prescription Knowledge questionnaire. With a negative predictive value of 82.2%, approximately one in every five patients would be classified as nonadherent, when they are truly adherent. The post-test probability of a negative result showed that when a diabetic patient has weak medical prescription knowledge, patient's probability of adherence reduces from 27% to 16%. Therefore most of the patients will not have good treatment adherence. A few adherent patients will be classified as nonadherent, these patients would unnecessarily be exposed to interventions to improve adherence. However, this would only serve to reinforce their adherence at relatively low cost.

Medical Prescription Knowledge questionnaire as proxy measurement of adherence facilitates the identification of a significant proportion of patients who do not adhere to the treatment, and who are candidates for interventions to improve adherence. This questionnaire provides physicians with information about the patient's knowledge of medications, dosage, and schedules, which could be responsible for a patient's nonadherence. This information enables physicians to intervene directly by modifying and/or reinforce patient's treatment knowledge, and eventually improve their adherence. It is known that in order to achieve satisfactory adherence, patients need to possess adequate knowledge about self-care behaviour [21]. In contrast, disease knowledge (not medical prescription knowledge), and self-report do not provide the information needed to adopt specific interventions.

### Reference to previous literature

Treatment adherence measured with pill count among our diabetic patients is low 27.0% when compared to 71% in the study of Mason [6] and that of Winkler 57.9%; although in the latter study the adherence dropped to 25%, when only patients who were prescribed two or three doses per day were considered [22]. Not only did we use the same method to measure adherence as they employed in both studies, but the definition of adherence applied here was also similar. However, these studies were restricted to patients who only received monotherapy, unlike the study carried out here. Polytherapy and doses prescribed two or three times per day negatively affect adherence [23], and in our study nearly 66% of the patients were prescribed two hypoglycemic drugs, and 88% were prescribed two or three doses per day of medication. Further, the population studied by Winkler [22] was observed over two months and they were voluntary patients of a specialized centre who might therefore have been more motivated. In Mexico, Duran-Varela et al. [24] identified an adherence of 54.2% using pill count as the measure of adherence, and over a time frame of 15 days; this short time period might result in an adherence overestimation.

Self-reports and questionnaires are widely used in clinical practice, and among the most outstanding are the Morisky-Green questionnaire [12] that evaluates adherence on the basis of patient's behavioural attitudes to treatment, and Batalla's questionnaire [9] that evaluates adherence on the basis of a patient's disease knowledge.

The Morisky-Green scale is designed to detect adherent patients [12]; this scale showed a specificity of 44% and a negative predictive value of 47%. Hence, it does not detect almost half the patients that are truly nonadherent. Piñeiro et al. [11] and García et al. [10] took nonadherence as the main event when they validated the Morisky-Green scale and they identified a sensitivity of 53.1 and 32.0%, respectively. Thus, from two-third to half of the nonadherent patients were misclassified as adherent.

When self-reporting is used to measure adherence, there are problems to identify nonadherent patients. When Haynes et al. [25] took adherence as a main event, they found 50% specificity, wrongly classifying half the patients that did not adhere. Similarly, when Piñeiro et al. [11] used nonadherence as the main event the sensitivity measured was 32.5%, wrongly classifying nearly two-third of nonadherent patients. In this latter study, Piñeiro also validated Batalla's questionnaire with nonadherence as the main event, defining a positive predictive value of 50.3% [11]. Thus, they classified almost half of the adherent patients as nonadherent, and half would be sent to an intervention program without needing it. In the studies of García et al., Piñeiro et al., and Haynes et al., [10,11,25] pill count was used as a gold standard measurement of adherence, which was defined as the consumption of 80–110% of the prescribed medication. This range tends to overestimate adherence, thereby wrongly classifying nonadherent patients as adherent.

### Limitations

The low adherence prevalence in the study population may have facilitated the good performance of Medical Prescription Knowledge questionnaire regarding the identification of nonadherent patients. Consequently, the questionnaire performance may change in contexts with higher adherence prevalence because its positive predictive value, positive likelihood ratio, and post-test probability for a positive result were low. Furthermore, the results cannot be generalized to all diabetic patients due to the fact that the study sample in this research did not have complications or insulin prescription. The presence of these characteristics could decrease adherence and affect sensitivity and specificity values.

### Suggestions for further research

As Medical Prescription Knowledge questionnaire performance may change in contexts with higher adherence prevalence, more research is needed before using this method in this kind of contexts. Moreover, this questionnaire should be tested through telephone interviews. It would explore the feasibility for covering more diabetic patients in order to detect risk people for non-adherence. It could be possible and cheaper because 75.5% of Mexican people have a lane line or a mobile phone [26].

As inadequate functional health literacy has been related with fewer years of education (≤ 6 years) in diabetic patients as well as with less knowledge of diabetes, including medications knowledge [27]; further research must measure health literacy along with medical prescription knowledge in order to improve adherence.

## Conclusion

Medical Prescription Knowledge questionnaire showed the best performance as proxy measurement to identify non-adherence in type 2 diabetic patients. The best performance was about negative predictive value, negative likelihood ratio, and post-test probability for a negative result. However, Medical Prescription Knowledge questionnaire performance may change in contexts with higher adherence prevalence. Therefore, more research is needed before using this method in other contexts.

## Competing interests

The authors declare that they have no competing interests.

## Authors' contributions

CAPA and YVM participated in the design of the study, data collection, statistical analysis, interpretation of data, and the manuscript draft. YSB collaborated in data collection, statistical analysis and manuscript draft. RRM participated in data collection and manuscript draft. RAU collaborated in statistical analysis and manuscript draft.

## Pre-publication history

The pre-publication history for this paper can be accessed here:



## Supplementary Material

Additional file 1 **Principal component analysis.** Attitude toward treatment adherence component loadings is presented for each item. Click here for file

Additional file 2 **Treatment adherence questionnaires.** The treatment adherence questionnaires provided are: Attitude toward Treatment Adherence and Medical Prescription Knowledge. Click here for file
