# Supplementary material for: Performance of two questionnaires to measure treatment adherence in patients with Type-2 Diabetes
Source: BMC Public Health. 2009 Jan 26;9:38. doi: 10.1186/1471-2458-9-38 (PMC2637241; doi:10.1186/1471-2458-9-38)
Supplement: Additional file 1 — Principal component analysis. Attitude toward treatment adherence component loadings is presented for each item. [file 1471-2458-9-38-S1.doc]

Attitude toward treatment adherence component loadings

| Item # | Factor 1 | Factor 2 | Factor 3 | Factor 4 | Factor 5 | Factor 6 |
| --- | --- | --- | --- | --- | --- | --- |
| AQ. 1 If diabetic patients feel well, they would stop taking their medications. | 0.65 |  |  |  |  |  |
| AQ. 2 Diabetic patients will get sicker if they stop taking their medications. | 0.64 |  |  |  |  |  |
| AQ. 3 In diabetic patients their medications will cause blindness. |  | 0.90 |  |  |  |  |
| AQ. 4 Diabetes is a disease that causes health complications. |  |  | 0.85 |  |  |  |
| AQ. 5 Medications for the treatment of diabetes will prevent or delay diabetes complications. |  |  | 0.88 |  |  |  |
| AQ. 6 For diabetic patients is difficult to take their medications at work. |  |  |  | 0.78 |  |  |
| AQ. 7 It is advisable that the diabetic patient’s family facilitates their intake of medications. |  |  |  | 0.57 |  |  |
| AQ. 8 Diabetic patients have problems complying with their treatment if they live far from the clinics. |  |  |  |  | 0.81 |  |
| AQ. 9 Diabetic patients have problems complying with their treatment due to lack of money. |  |  |  |  | 0.82 |  |
| AQ. 10 Physicians and diabetic patients should agree with the diabetes prescriptions. |  |  |  |  |  | 0.64 |
| AQ. 11 Do you agree with your diabetes treatment? |  |  |  |  |  | 0.85 |
