# Supplementary material for: Performance of two questionnaires to measure treatment adherence in patients with Type-2 Diabetes
Source: BMC Public Health. 2009 Jan 26;9:38. doi: 10.1186/1471-2458-9-38 (PMC2637241; doi:10.1186/1471-2458-9-38)
Supplement: Additional file 2 — Treatment adherence questionnaires. The treatment adherence questionnaires provided are: Attitude toward Treatment Adherence and Medical Prescription Knowledge. [file 1471-2458-9-38-S2.doc]

Attitude Toward Treatment Adherence Questionnaire

For each of the following questions we invite you to review the answer sheet and respond according to what best represents your view toward the statement presented in the question.

With the following 11 questions we wish to know to what extent you agree or disagree with the situation that helps or limits diabetic patients with their treatment compliance as indicated by physicians

|  | Strongly agree | Agree | Neither agree or disagree | Disagree | Strongly disagree | Score |
| --- | --- | --- | --- | --- | --- | --- |
| AQ. 1 If diabetic patients feel well, they would stop taking their medications. (-) | 1 | 2 | 3 | 4 | 5 |  |
| AQ. 2 Diabetic patients will get sicker if they stop taking their medications (+) | 5 | 4 | 3 | 2 | 1 |  |
| AQ. 3 In diabetic patients their medications will cause blindness  (-) | 1 | 2 | 3 | 4 | 5 |  |
| AQ. 4 Diabetes is a disease that causes health complications (+) | 5 | 4 | 3 | 2 | 1 |  |
| AQ. 5 Medications for the treatment of diabetes will prevent or delay diabetes complications (+) | 5 | 4 | 3 | 2 | 1 |  |
| AQ. 6 For diabetic patients is difficult to take their medications at work (-) | 1 | 2 | 3 | 4 | 5 |  |
| AQ. 7 It is advisable that diabetic patient’s family facilitates their intake of medications (+) | 5 | 4 | 3 | 2 | 1 |  |
| AQ. 8 Diabetic patients have problems complying with their treatment if they live far from the clinics (-) | 1 | 2 | 3 | 4 | 5 |  |
| AQ. 9 Diabetic patients have problems complying with their treatment due to lack of money (-) | 1 | 2 | 3 | 4 | 5 |  |
| AQ. 10 Physicians and diabetic patients should agree with the diabetes prescriptions (+) | 5 | 4 | 3 | 2 | 1 |  |
| AQ. 11 Do you agree with your diabetes treatment? (+) | 5 | 4 | 3 | 2 | 1 |  |
| TOTAL SCORE |  | | | | |  |

Medical Prescription Knowledge Questionnaire

From the medical record of the diabetic patient, the family physician’s prescription must be identified. Check (**√**) the corresponding box if the statement corresponds to the information written in the medical record of a diabetic patient.

Medication prescribed A: Glibenclamide dosage:

1 tablet at each intake

2 tablets at each intake

Glibenclamide dosage frequency:

Medication once a day:

Medication twice a day:

Medication three times a day:

Medication prescribed B: Metformin dosage:

1 tablet at each intake:

2 tablets at each intake:

Metformin dosage frequency:

Medication once a day:

Medication twice a day:

Medication three times a day:

With the following three questions we wish to know how you take the medication prescribed by your family physician at the last medical visit. If the patient´s answer corresponds with the information listed in the last section check (**√**) the corresponding box of the statement, otherwise place an (**x**). If the medication is not prescribed, leave the box blank.

KQ. 1. What is the name of the diabetes medication prescribed by your family physician?

Medication A: Glibenclamide:

Medication B: Metformin:

KQ. 2. How many tablets a day do you have to consume at each intake?

Medication A: Glibenclamide:

1 tablet at each intake:

2 tablets at each intake:

Medication B: Metformin:

1 tablet at each intake:

2 tablets at each intake:

KQ. 3. How many times a day do you have to take your medication?

Medication A: Glibenclamide:

Once a day:

Twice a day:

Three times a day:

Medication B: Metformin:

Once a day:

Twice a day:

Three times a day:
